# “MYH9 mutation and squamous cell cancer of the tongue in a young adult: a novel case report”

**DOI:** 10.1186/s13000-022-01210-x

**Published:** 2022-02-06

**Authors:** Takako Eva Yabe, Kylie King, Susan Russell, Laveniya Satgunaseelan, Ruta Gupta, James Chen, Bruce Ashford

**Affiliations:** 1 Department of Head and Neck Surgery, Wollongong Hospital, Wollongong, NSW Australia; 2 Illawarra Health and Medical Research Institute, Wollongong, NSW Australia; 3 Illawarra Shoalhaven Local Health District, NSW Health Pathology South, Wollongong Hospital, Wollongong, NSW Australia; 4 Kids Cancer Centre, Sydney Children’s Hospital, Sydney, NSW Australia; 5 School of Women’s and Children’s Health, University of New South Wales Sydney, Sydney, NSW Australia; 6 Department of Tissue Pathology and Diagnostic Oncology, Royal Prince Alfred Hospital, Sydney, NSW Australia; 7 Central Clinical School, The University of Sydney, Sydney, NSW Australia; 8 Department of Radiation Oncology, Wollongong Hospital, Wollongong, NSW Australia; 9 Faculty of Science, Medicine and Health, University of Wollongong, Wollongong, NSW Australia

**Keywords:** Squamous cell carcinoma, Tongue, Young adult, *MYH9*, Epstein syndrome, Neck dissection, Free flap, Microvascular surgery

## Abstract

**Background:**

The incidence of tongue cancer in young adults is on the rise. This trend is more pronounced in females. Although the aetiology is still unclear, there is mounting evidence that genetic syndromes can play a key role in development of oral cancers in this patient group. We report the first case of oral squamous cell carcinoma (oSCC) in a young adult with an MYH9-related disorder (MYH9-RD).

**Case presentation:**

A 19-year-old female with a germline *MYH9* variant (missense variant in exon 2: c.287C > T, (p.Ser96Leu)) was referred to the head and neck surgery department for investigation of a painful, thick right tongue ulcer. She was diagnosed with Epstein syndrome, an MYH9-RD, at 12 years of age. Her main phenotypic features were profound thrombocytopenia and marked renal impairment. The tongue biopsy confirmed SCC. Preoperative positron emission tomography (PET) revealed avidity in the right tongue and ipsilateral level 2A neck lymph node. With substantial preoperative multidisciplinary input, she underwent cancer ablation and microvascular free flap reconstruction. Her pathology showed a 35 mm diameter, 14 mm thick moderately differentiated SCC with perineural and lymphovascular invasion. Two out of 38 right neck nodes were positive for metastasis with extranodal extension. None of the 34 left neck nodes was involved.

She had an uneventful recovery and was discharged home on day 6 postoperative day. On day 15 postoperative day, she had catastrophic bleeding in the neck with a respiratory arrest after a forceful cough. She required an emergency tracheostomy and returned to the theatre for haemostasis. Following a short inpatient stay, she was again discharged home and underwent adjuvant therapy consisting of external beam radiotherapy of 60Gy in 30 fractions. On clinical examination and PET at 6 months after surgery, she had no evidence of disease recurrence.

**Conclusions:**

MYH9-RD can present with advanced locoregional oral cavity malignancy at an early age. The combination of profound thrombocytopenia and marked renal impairment can impact heavily on routine major head and neck cancer surgery and adjuvant treatment. This rare and challenging condition underlines the importance of early detection of cancer and multidisciplinary team input.

## Background

An international multi-institutional analysis by Satgunaseelan et al. [1] demonstrated that the incidence of tongue cancer is rising faster in younger patients (≤ 45 years of age) compared to older patients (> 45 years of age). In the younger cohort, the incidence increased at significantly higher rate in females, compared to male (4.3% vs 1.5% per year). There are certain genetic syndromes such as Fanconi anaemia [2] and dyskeratosis congenita [3], which are known to be linked to oral cancers at an early age. To date, there is no report of a tongue cancer in a young adult with Myosin Heavy Chain 9 (*MYH9)* mutation.

The *MYH9* gene encodes the non-muscular myosin heavy chain IIA (NMHCIIA), a cytoskeletal contractile protein. The NMHCIIAs are expressed in all eukaryotic cells [4]. *MYH9* mutation may result in thrombocytopaenia and platelet macrocytosis, sensorineural deafness, cataracts and/or renal impairment. These rare autosomal dominant disorders are known as MYH9-related disorders (MYH9-RDs), and encompass May-Hegglin anomaly, Sebastian, Fechtner, and Epstein syndromes [5]. MYH9-RDs are rare, and only 113 unrelated families are reported in the literature [6]. Based on the Exome Aggregation Consortium (ExAC) database, prevalence is approximately 1:20,000 – 25,000 [7]. The expression of clinical features is heterogenous [8]. In some individuals, the haematological features remain the only manifestations of the disorder throughout life. It is thought that approximately 35% of index cases are sporadic [9, 10]. In more than half of these cases, a de novo mutation was confirmed by segregation analysis.

Besides the motorised protein impacts, differential expression of *MYH9* has been implicated in cancer invasion and metastasis [11–14]. *MYH9* can act both as an oncogene and a tumour suppressor in different cancers; however, in the context of head and neck cancers, *MYH9* has been shown to have synergistic influence on cell invasion with *TP53* [15]. *TP53* is the most frequently mutated gene in head and neck squamous cell carcinoma (HNSCC), occurring in more than 70% of cases of non-human papillomavirus (HPV) SCC [16–18]. Most high impact alterations involving tumour suppressor genes render them non-functional through truncation or deletion [15]. However, *TP53* is unique because there is a strong propensity for missense mutations, particularly within its DNA-binding domain. *MYH9* mutations have been shown to impair an in vitro p53 response to DNA damage, with the majority of *MYH9* mutations clustering in the ATPase domain [13]. This tumour suppressive role of *MYH9* is enacted through NMHCIIA [15]. Cell line studies have shown that inhibition of NMHCIIA prevents the nuclear localisation of p53, crucial for downstream tumour suppressive functions [13, 15, 19–21]. The tumour suppressor capability of p53 is therefore dependent on NMHCIIA function in head and neck cancer. Inhibition of NMHCIIA leads to increased invasion in wildtype (wt)p53 expressing cells but not in high-risk mutated (mut)p53 expressing cells [15]. These findings indicate that cells with a wtp53 phenotype are dependent on NMHCIIA inhibition for invasive capability, secondary to decreased nuclear accumulation of wtp53, and subsequent reduction in target gene expression. In contrast, cells harbouring high-risk mutp53 attain an invasive phenotype independent of NMHCIIA expression [17].

*MYH9* is a well-conserved gene through evolution from fungi to mammals. The similarity of human *MYH9* to that of mice in the genomic organisation suggests that the mouse is an excellent model to study the pathogenetic mechanisms responsible for human disease [22]. A homozygous NMHCIIA*-*knockdown mouse model demonstrated development of tongue SCC [23]. SCC occurred very early during embryonic epithelium development, with a 100% penetrance. Similar to the aforementioned cell line studies, oncogenesis in this mouse model was independent of defective p53 activation. From a protein perspective, Schramek et al. (2014) found that 24 and 31% of human skin and head and neck SCCs respectively were characterised by negligible or weak NMHCIIA immunohistochemical expression [13]. There is therefore accumulating evidence that *MYH9* and its expression at both RNA and protein levels might play an important role in HNSCC.

## Case presentation

A 19-year-old non-smoker female patient with *MYH9* mutation (missense variant in exon 2: c.287C > T, (p.Ser96Leu)) was referred to our department for investigation of a painful, thick right tongue ulcer with reactive lymphadenitis. She had been diagnosed with oral thrush but had not responded to antifungal therapy. Her symptoms had persisted for some months. Her assessment was compromised by the unavailability of face to face consultation with her general practitioner during the early days of the Covid-19 pandemic.

**Fig. 1:**
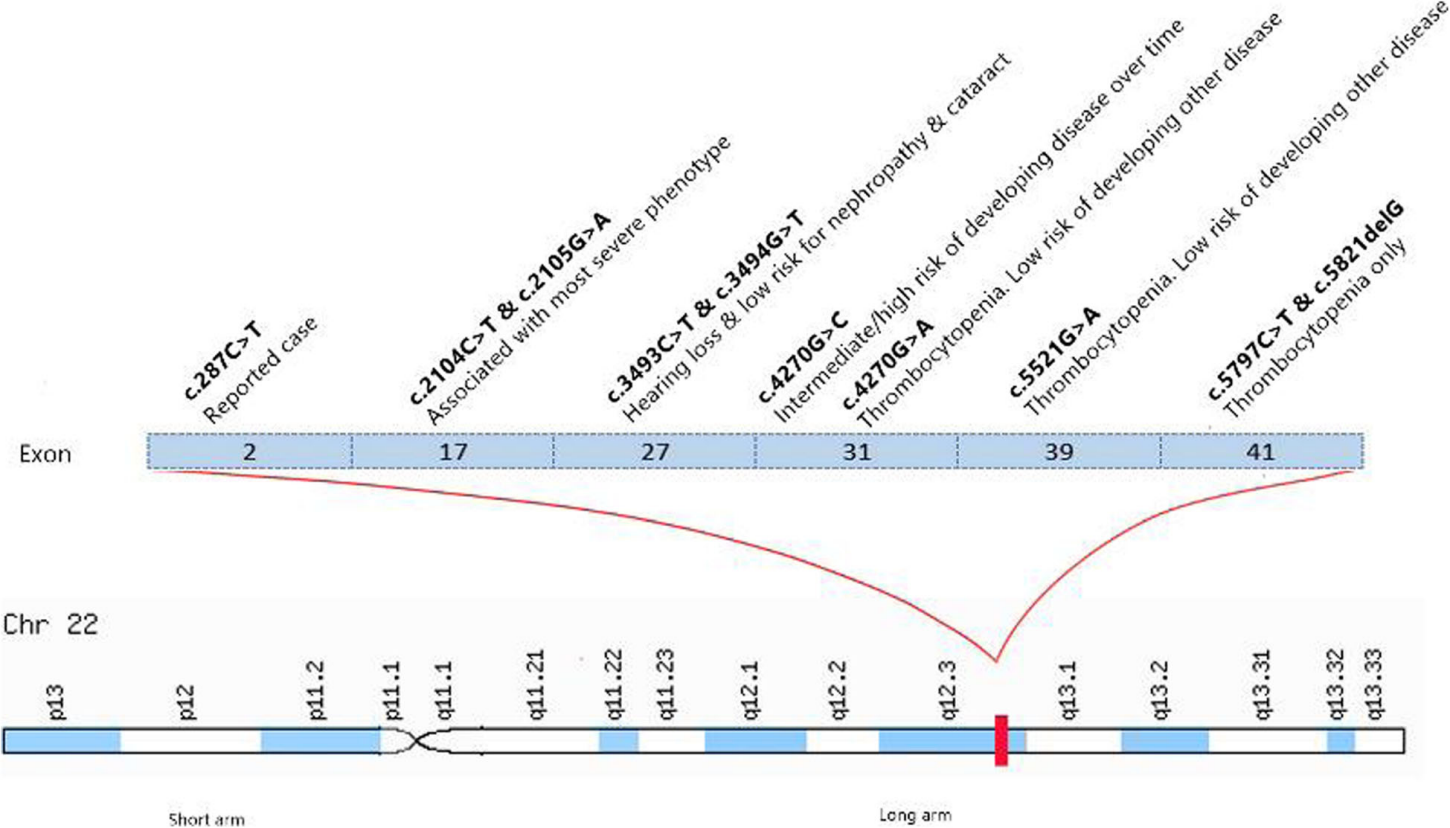
*MYH9* exons and mutations. Notable *MYH9* pathogenic variants described by Savoia and Pecci (2021) [24]

She was diagnosed with Epstein syndrome at 12 years of age with severe thrombocytopenia (baseline count ~ 5 × 10^9^/L), nephrotic syndrome with proteinuria (creatinine 298 with eGFR 19), and sensorineural deafness. When she was 18 months old, she was referred to a tertiary paediatric hospital by a local paediatrician for a sudden onset of bruising and a platelet count of 13 × 10^9^/L. The giant platelets were seen on blood films. Her parents both had normal platelet counts and blood films. The only family history was her maternal great-aunt, who died of leukaemia at 40 years of age. She was commenced on treatment for acute idiopathic thrombocytopenic purpura (ITP). A bone marrow aspirate and trephine were performed when she turned 7 years old. The trephine showed a normocellular marrow with increased megakaryocytes consistent with peripheral destruction. Cytogenetics confirmed a 46XX karyotype. She developed a life-threatening epistaxis when she was 8 years old, for which she was resuscitated with a pack red cell and platelet transfusion. She received intravenous immunoglobulin (IVIG) then, which was complicated by aseptic meningitis. Her platelet count rose from 1 to 87 × 10^9^/L, which was likely due to the platelet transfusion rather than the IVIG. She remained well until she was 12 years old when she had profound menorrhagia with haemoglobin (Hb) of 55 g/L and platelets of 4 × 10^9^/L. again she was administered blood product replacement as well as commenced on medroxyprogesterone and prednisolone 4 mg/kg/day for the provisional diagnosis of chronic ITP. The maximal platelet count during this admission was 38. This was likely due to transfusion rather than prednisolone. Following this episode, she participated in the multi-centre international randomized controlled trial of romiplostim versus placebo in children with chronic ITP (Amgen study 20,080,279). Pre-trial screening incidentally revealed 3+ proteinuria. Her serum albumin, creatinine and blood pressure were all normal. Her DNA was sent to Bristol Genetics Laboratory for steroid-resistant nephrotic syndrome gene panel testing. Analysis of the *MYH9* gene revealed a pathogenic heterozygous missense mutation in exon 2, c.287C > T, (p.Ser96Leu), which confirmed the diagnosis of MYH9-RD (Fig. 1). Her phenotype was that of autosomal dominant Epstein Syndrome.

Once the diagnosis of MYH9 related disorder diagnosis was confirmed, she withdrew from the trial. Later it was notified that she was receiving romiplostim, not a placebo. Since she was 18 months old, the highest recorded platelet count was 86 × 10^9^/L when she received six weeks of 2 mg/kg/day prednisolone and was up to 9 mcg/kg/dose per week of romiplostim concurrently.

**Image 1:**
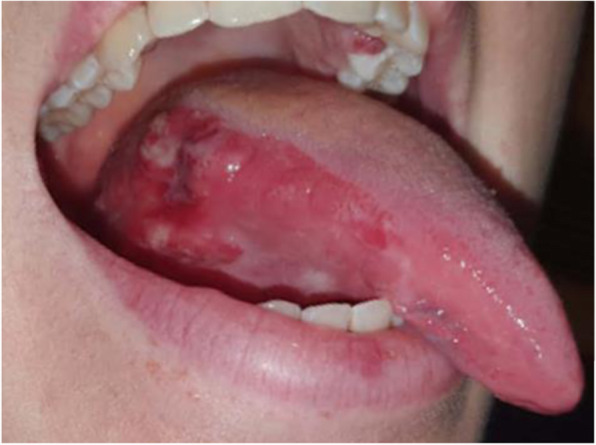
Clinical photograph of right tongue lesion at presentation shows an ulcerated tumour with surrounding oedema and erythroplakia. Inflammatory/haemorrhagic lesion on the palatal aspect of left maxillary premolar was also noted

At initial surgical consultation, the patient reported a non-healing, painful, tongue ulcer unrelated to any trauma. She noticed the ulcer eight months prior, but unfortunately, the presentation was delayed due to COVID-19 and its surrounding safety measures. By the time of presentation, the ulcer has grown to 4 cm in size (Image 1).

**Image 2:**
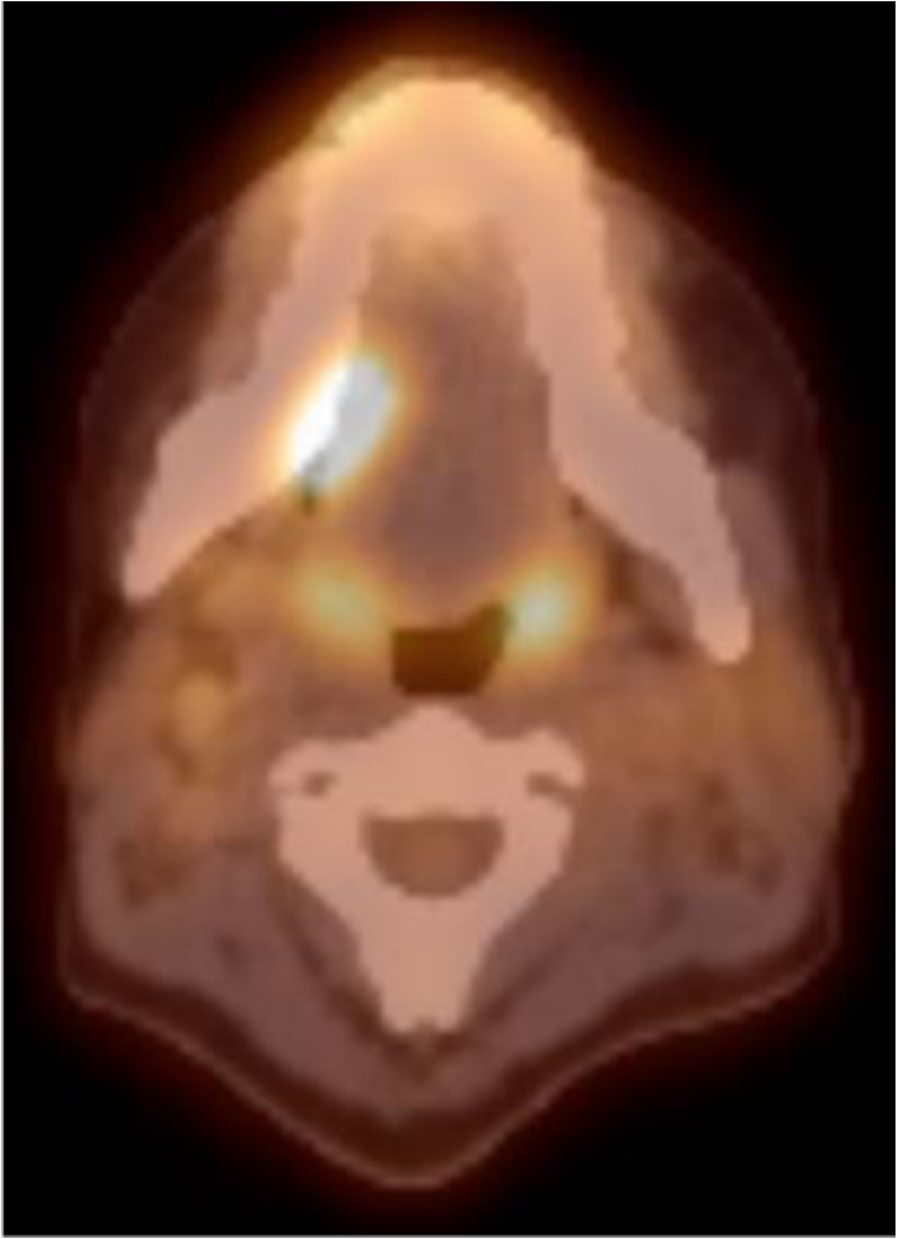
Pre-operative PET/CT demonstrating 2,3 fluorodeoxyglucose avidity in the right tongue tumour. Mild lymph node avidity was also demonstrated but no evidence of distant PET avid disease

Point of care ultrasound revealed lymphadenopathy. The working provisional clinical diagnosis was SCC. Tissue biopsy required preoperative platelet transfusion. The biopsy confirmed SCC in the primary site, but no malignancy was found in the palpable ipsilateral neck lymph nodes. Computed tomography (CT)/FDG PET showed suspicious avidity in the right tongue and 2A cervical node (Image 2).

Following extensive multidisciplinary consultation and planning, she underwent surgery which comprised tracheostomy, right hemiglossectomy, third molar tooth extraction, bilateral neck dissection (right level 1–4, left level 1–3), and radial forearm free flap reconstruction with microvascular anastomosis. Radial forearm free flap with 4 by 6 cm fasciocutaneous paddle was harvested with a vascular pedicle consisting of the radial artery, venae commitantes, and the cephalic vein. They were anastomosed to superior thyroid artery, internal jugular vein and common facial vein, respectively. Vessel diameters were between 2 and 4 mm. Polyglactin 910 (Vicryl™; Ethicon) 3–0 suture was used for the inset of the free flap to intraoral defect, and Vicryl™ 3–0 and poliglecaprone (Monocryl™; Ethicon) 4–0 sutures were used for skin closure. She received 2 HLA matched pool platelets perioperatively aiming for the count above 50 × 10^9^/L. One unit of packed red cell and 2 g of tranexamic acid were administered intraoperatively. The surgical procedure was completed with a total operative time of 6 h 42 min with an ischaemic time of 1 h 20 min for the free flap harvest. The tourniquet time was 58 min. The estimated total blood loss was 200 mL. The platelet count was 69 × 10^9^/L, and Hb was 94 g/L at the completion of the case. She received routine postoperative orders, including monitoring in the high dependency unit (HDU), hourly flap observations, 48 h of intravenous (IV) antibiotics (cephazolin 1 g three times per day and metronidazole 500 mg twice per day), nasogastric feeding, but no chemical venous thromboembolism (VTE) prophylaxis. She had an uneventful recovery including the removal of her tracheostomy on day 4 and was discharged home on day 6 postoperative day on a soft diet.

On day 15, she was assessed by the speech and language pathologist. Part of this assessment was a modified barium swallow study. During this test she had a coughing paroxysm. This precipitated neck swelling and airway embarrassment. She was transported to the Emergency Department for urgent assessment. Due to impending respiratory arrest, an emergency airway was established via the existing tracheostomy site which had not fully healed and the patient was then transferred to theatre for neck exploration and control of haemorrhage. Bleeding was found from the free flap pedicle vessels. After two days of intensive care unit (ICU) then surgical ward stay, she was discharged home with surgical and radiation oncology follow up.

**Image 3:**
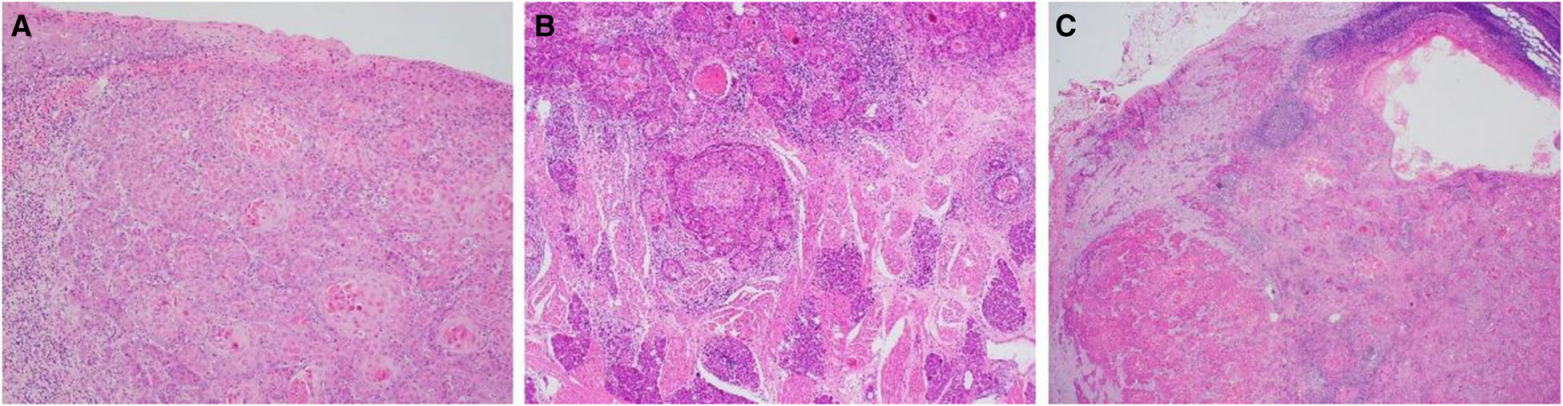
Histological slides **a** SCC (H&E × 100) at surface of tumour demonstrating irregular nests of highly atypical squamous cells invading the submucosal space, with adjacent oral mucosa at top left; **b** SCC (H&E × 100), present as infiltrative tumoural nests with keratinisation, at invasive front of tumour; **c** Lymph node metastasis (H&E × 20) demonstrating extranodal extension of SCC in to adjacent soft tissue

Her pathology demonstrated a moderately differentiated SCC of the oral tongue with a maximum dimension of 35 mm and up to 14 mm thick. The tumour had an endophytic growth pattern, and multiple foci of lymphovascular invasion (LVI) and perineural invasion (PNI) were seen. Margins were clear. Two (level IB and IIA) out of 38 right neck nodes contained metastatic SCC, measuring 22 mm, and both had a focal extranodal extension of 1 mm (Image 3). None of the 34 lymph nodes from the left neck was involved. Her pathological tumour stage was Stage 4 (pT3, pN3b), according to the American Joint Committee on Cancer (AJCC) 8th edition.

**Image 4:**
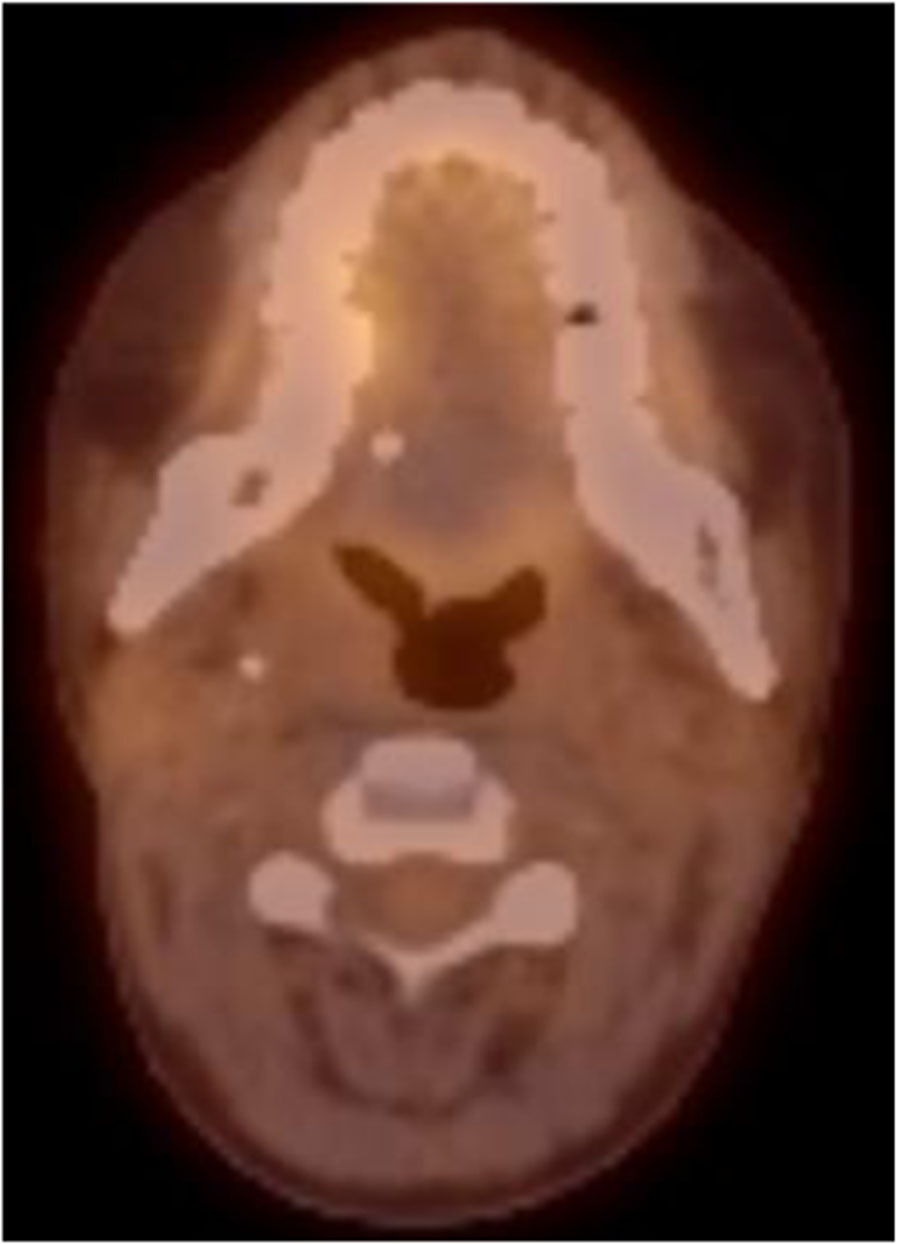
Post-operative PET/CT at 6 months showing mild persistent avidity in the right tongue in keeping with local trauma to the flap reconstruction

She had clear indications for adjuvant chemoradiotherapy, including nodal involvement, extracapsular extension and multifocal LVI and PNI. She received adjuvant radiotherapy but, despite disease indications, was unable to receive chemotherapy due to her renal impairment. She received 60Gy RT in 30 fractions. On clinical examination and PET at 6 months after surgery she had no evidence of disease recurrence (Image 4).

## Discussion and conclusions

Tongue cancers in young adults, let alone teenagers, are relatively uncommon compared to the older individuals. Time to diagnosis may also be longer because healthcare professionals may not have thought about malignancy as differential diagnosis. Retrospective analysis of adolescent and young adult generation with oral cancer by Yoshioka et al. [25] demonstrated that tongue was the most common subsite and SCC was the most common histological type. The role of MYH9 in oSCC in humans has not been described before, however, various studies have shown in vitro tumorigenic properties of MYH9 variation [26–29]. The role of *MYH9* in cancer is context dependent and can act both as a tumour promoter and suppressor in different cancers. The role of *MYH9* as a tumour suppressor has been proposed in murine models of head and neck cancer, where loss of *MYH9* results in the development of SCC and increased cell invasion [15, 23]. In the reported case, the combination of genetic predisposition and immunosuppression by renal failure further increases the risk of HNSCC. Murayama et al. (2013) reported a case of 1 year old boy and 33 year old father who shared the same genetic mutation as the reported case [30]. Their clinical course was similar to the case presented except neither developed documented malignancy.

The observation of an affected family pedigree described in the paper by Althaus (2010) demonstrated the following; 1) bleeding is usually not the leading problem in many of these patients 2) in patients with a history of only minor bleeding, prophylactic platelet transfusion before the major surgery is not necessary 3) pregnancy is usually not complicated by significant bleeding and spontaneous delivery seems to be appropriate without risk of intracerebral bleeding as newborns 4) MYH-9 related thrombocytopenia does not protect from a thromboembolic event such as myocardial infarction (MI). Around 28% of patients present spontaneous mucosal bleeding, mainly menorrhagia, epistaxis, and gum bleeding, but life-threatening haemorrhages are rare [31]. In the presented case, she had life-threatening haemorrhages since her childhood and required perioperative platelet transfusion prophylactically.

A low haematocrit will exacerbate the impaired platelet subendothelial interactions even with normal platelets [32]. The thrombopoietin receptor agonist Eltrombopag has been used successfully for preparing patients with severe MYH9-related thrombocytopenia for elective surgery [33–35]. Romiplostim, another agonist of the thrombopoietin receptor, was also used for this purpose in one MYH9-RD patient [36]. Thrombopoietin receptor agonists increase the production of even larger platelets because the fragmentation defect is still present. These giant platelets are larger than the capillaries’ diameter, and increasing the number of giant platelets may worsen the microcirculation [6]. Therefore, thrombopoietin receptor agonist could be considered as an adjunct to major surgery, however, its effect on microcirculation needs to be carefully considered in free flap reconstructions.

The aims of cancer care are histological confirmation of diagnosis, staging of disease and definitive treatment. Each step of the management in this case was impacted by the underlying bleeding tendency and renal failure. For diagnosis of oSCC, a punch or incisional biopsy is required. In the case of palpable cervical lymphadenopathy, cytological assessment by ultrasound guided FNA is also indicated. These procedures can usually be performed as an outpatient under local anaesthesia. However, in this case, the patient required preoperative platelet transfusion, general anaesthesia and overnight inpatient observation to safely manage the risk of airway obstruction due to bleeding. Standard staging protocol for oSCC includes CT head and neck, or magnetic resonance imaging (MRI), and PET scan. The purpose of imaging is to assess local staging and distant disease. The presence of distant disease precludes the patient from having a curative surgery. The imaging enhancing contrast may be nephrotoxic [37]. Renal protective measures such as intravenous (IV) fluid and N-acetylcysteine may be required.

Standard therapy for locally advanced lateral tongue SCC is partial glossectomy, selective neck dissection and reconstruction. This patient underwent bilateral instead of ipsilateral neck dissection as the tumour was clinically thick, and there was a clinical concern for bilateral disease. Tracheostomy was created for airway protection in case of large haematoma or life-threatening haemorrhage. Tracheostomy is not routinely performed for the size and location of her resection. She was decannulated on day 2 postoperative day. Reconstruction using free tissue transfer is routine following hemiglossectomy in order to achieve acceptable tongue function, swallowing and speech.

The use and type of postoperative anticoagulation is institution dependent. At least two patients with MYH9-RDs are reported to have developed thrombosis following surgery [38, 39]. Therefore venous thromboembolic prophylaxis should be considered in situations with a high risk of thrombosis. It is crucial if antifibrinolytic agents are given to prevent bleeding during the surgery. The application, indications, timing, and duration are currently based on personal experience and practice [40]. However, most institutions use subcutaneous heparin or low molecular weight heparin (LMWH). Because vascular autonomic regulation is often impaired by the flap harvest significantly higher perfusion values can be observed at the time of subsequent reperfusion [41]. Heat-induced vasodilatation, which requires intact innervation (mostly mediated by c-afferents) and endothelial function, is also lost in the free flaps [42, 43]. Surgical manipulation of the endothelium and the trauma often leads to anastomotic complications due to the sub-intimal collagen exposure; moreover, it triggers coagulation and microthrombi formation [44]. Hypercoagulability, venous stasis, and endothelial injury, known as Virchow’s triad, are all encountered during free flaps surgery [45]. It is uncertain if the presence of giant platelets compromises microcirculation within free flaps due to altered viscosity. Preoperative chemical VTE prophylaxis was omitted in our patient while mechanical VTE prophylaxis, including thromboembolic deterrent stocking (TEDS) and intermittent pneumatic compression (IPC), were applied. The decision was based on the assumption that she would have lower risk of VTE.

When this patient was taken back to theatre for life-threatening bleeding after a forceful coughing during swallow assessment, the feeding vascular pedicle was ligated. The free flap survived despite early loss of feeding vessels. There is no data available for tissue healing in the setting of *MYH9* mutation.

The platelet count was 8 × 10^9^/L on the modified barium swallow test. The blood count is not routinely checked before postoperative functional studies. We suggest to include routine haematological examination before post-operative swallowing assessments. As described in our case, a forceful coughing during routine swallow test induced life-threatening haemorrhage and respiratory arrest. Peri-assessment platelet transfusion may be necessary at discretion of treating haematologist.

Typically, in the setting of extranodal extension of SCC, combination chemoradiation with cisplatin is indicated to improve regional control. However chemotherapy was omitted in this case due to the pre-existing thrombocytopenia, impaired renal function and impaired hearing. All patients undergoing head and neck radiation treatment develop confluent mucosal ulcers as well as skin desquamation. Basic supportive measures are generally sufficient, but these ulcers may bleed significantly in this patient’s situation, and such problems can persist for several weeks. To prevent catastrophic bleeding during radiotherapy treatment, she needed to receive regular platelet transfusion during the second half of the treatment regime and 3 to 4 weeks post-treatment.

This patient had extensive ablative and reconstructive surgery as she presented with locally advanced disease. If the primary disease were detected early, it would have been possible to manage it with a less invasive and less morbid procedure such as laser excision. It emphasises the importance of education to the patient, family and treating clinicians to have a low threshold to investigate suspicious idiopathic lesions.

The usual time frame for head and neck cancer surveillance is five years. There is no reliable data on risk of second primary oral cavity malignancy, and so surveillance may need to be prolonged.

We report a 19 year old female patient with Epstein syndrome with a locally advanced tongue SCC who underwent extensive ablative and reconstructive surgery. This is the first case of oral tongue SCC in the context of MYH9-RD. Complex multidisciplinary consultation and planning were the cornerstones of safe patient care. The importance of cancer awareness and early detection in MYH9-RD is emphasised and oral cavity SCC in these diseases requires further characterisation.

## Data Availability

Data sharing is not applicable to this article as no datasets were generated or analysed during the current study. Not applicable.

## Abbreviations

CT: computerised tomography
FDG/PET: Fluorodeoxyglucose/positron emission tomography
FNA: fine needle aspirate
HB: haemoglobin
HLA: human leukocyte antigen
ICU: intensive care unit
IPC: intermittent pneumatic compression
ITP: thrombocytopenic purpura
IVIG: intravenous immunoglobulin
LMWH: low molecular weight heparin
LVI: lymphovascular invasion
MRI: magnetic resonance imaging
MUT: mutated
*MYH9*: Myosin Heavy Chain 9
NMHCIIA: non-muscular myosin heavy chain IIA
oSCC: oral squamous cell carcinoma
PNI: perineural invasion
TEDS: thromboembolic deterrent stocking
VTE: venous thromboembolism
WT: wild type
